# Determinants of Dwell Time in Visual Search: Similarity or Perceptual Difficulty?

**DOI:** 10.1371/journal.pone.0017740

**Published:** 2011-03-08

**Authors:** Stefanie I. Becker

**Affiliations:** School of Psychology, The University of Queensland, Brisbane, Queensland, Australia; Macquarie University, Australia

## Abstract

The present study examined the factors that determine the dwell times in a visual search task, that is, the duration the gaze remains fixated on an object. It has been suggested that an item’s similarity to the search target should be an important determiner of dwell times, because dwell times are taken to reflect the time needed to reject the item as a distractor, and such discriminations are supposed to be harder the more similar an item is to the search target. In line with this similarity view, a previous study shows that, in search for a target ring of thin line-width, dwell times on thin linewidth Landolt C’s distractors were longer than dwell times on Landolt C’s with thick or medium linewidth. However, dwell times may have been longer on thin Landolt C’s because the thin line-width made it harder to detect whether the stimuli had a gap or not. Thus, it is an open question whether dwell times on thin line-width distractors were longer because they were similar to the target or because the perceptual decision was more difficult. The present study de-coupled similarity from perceptual difficulty, by measuring dwell times on thin, medium and thick line-width distractors when the target had thin, medium or thick line-width. The results showed that dwell times were longer on target-similar than target-dissimilar stimuli across all target conditions and regardless of the line-width. It is concluded that prior findings of longer dwell times on thin linewidth-distractors can clearly be attributed to target similarity. As will be discussed towards the end, the finding of similarity effects on dwell times has important implications for current theories of visual search and eye movement control.

## Introduction

The factors determining our eye movement behaviour have been studied extensively in a wide range of contexts. One of the most extensively studied paradigms is the visual search task, where observers have to search for a pre-specified target (e.g., a particular letter or shape) among irrelevant distractors, and to indicate the presence or absence of the target by pressing a key [1]. Whereas much research has been devoted to investigating the factors that modulate the number of fixations needed to find the target, much less is known about the factors that modulate dwell times (i.e., the duration the gaze lingers on individual stimuli) – perhaps, because it has been shown that search efficiency, or the overall time needed to find the target, is much more closely correlated with the number of fixations than with dwell times [2]–[3].

One determinant of dwell times might be the similarity of items to the to-be-searched for target. For instance, in one study [4], the effect of target similarity on dwell times was examined when observers had to search for a ring among Landolt Cs (i.e., rings with a small gap). The target ring always had thin line-width, and dwell times were separately assessed for distractors that were similar to the target (i.e., thin line-width distractors) and those that were more dissimilar from the target (i.e., distractors with medium and thick line-width). The results showed that dwell times on the target-similar, thin Landolt C’s were significantly longer than dwell times on the medium and thick Landolt C’s. Correspondingly, it was concluded that dwell times are determined by the similarity of the stimulus to the target [4].

However, it should be noted that the distractors also differed with respect to their *perceptual difficulty*, as it may be generally harder to detect a gap in a thin Landolt C than in a thick Landolt C. To assess this possibility, a pilot study (n = 5) was conducted where observers had to respond to a centrally presented (50 ms) Landolt C or closed ring in a gap detection task. The results showed that thin Landolt C’s produced significantly slower RT (M = 383 ms) than medium and thick Landolt C’s (medium: M = 369 ms; *t*(4) = 4.6; *p* = .010; thick: M = 369 ms; *t*(4) = 4.5; *p* = .011). The same trends could also be observed in the mean error scores, with more errors in the thin condition (9.6%) than in the medium and thick conditions (4.7%; *t*(4) = 2.6; *p* = .061, and 6.6%, *t*(4) = 2.4; *p* = .078, respectively), indicating that detecting the gap in the thin Landolt C was more difficult than detecting it in the medium and thick Landolt C’s.

These results indicate that longer dwell times on thin Landolt C’s might be due to differences in perceptual difficulty, and not target similarity [5]–[7]. – In line with this hypothesis, previous studies found an effect of perceptual difficulty on dwell times [8]: For instance, when observers have to search for a letter in an array of 10×10 letters, increasing the luminance contrast of all stimuli significantly decreased mean dwell times, from 251 ms in the lowest contrast condition, to 194 ms in the highest contrast condition [8]–[10]. Since changing the luminance contrast would not have affected target-distractor similarity, elongated dwell times in the low contrast condition were probably due to an increase in the difficulty of the perceptual decision.

It is important to disentangle such effects of perceptual difficulty from similarity, because arguably, they reflect impacts of different kinds of processes: Whereas differences in perceptual difficulty could modulate dwell times in a purely bottom-up, stimulus-driven manner, discovering an effect of target similarity would show an effect of top-down search strategies on dwell times [11]. The aim of the present study was to decouple perceptual difficulty from target-distractor similarity, measuring dwell times in a visual search experiment similar to the previous study that allegedly found that similarity affected dwell time [4]. Unlike previous studies, target similarity was varied independently of perceptual difficulty, by including 3 blocked conditions which included targets of varying line-width: thin, medium or thick.

If dwell times are predominantly modulated by the perceptual difficulty of detecting a gap in stimuli of different line-widths, then we would expect dwell times on distractors with thin line-width to be consistently longer than dwell times on the medium or thick distractors, regardless of their similarity to the target. In contrast, if dwell times are primarily modulated by similarity, then we would expect dwell times to vary with the target type, resulting in longer dwell times on distractors that are similar to the target than on distractors that are dissimilar from the target.

## Methods

### Participants

12 students from The University of Queensland, Australia (5 male, 7 female, mean age: 28.5), took part in the experiment as paid volunteers ($10). The present study was conducted in accordance with the ethical principles expressed in the Declaration of Helsinki, and the study has been approved by the ethics committee of the University of Queensland as complying with the regulations of the National Statement on Ethical Conduct in Human Research. All participants provided informed, written consent to participate in the study Materials. The Software Presentation (Neurobehavioral Systems), run on an Intel Duo 2 CPU 2.4 GHz computer with a 17” LCD monitor (resolution: 1280 * 1024; 75 Hz vertical refresh), was used to present the stimuli. For eye tracking, a video-based eye tracker with a spatial resolution of 0.1° and a temporal resolution of 500 Hz was used (Eyelink 1000, SR Research, Ontario, Canada). Participants viewed the screen from a distance of 57 cm, and responded by clicking one of two buttons of a standard USB mouse.

### Stimuli

**Figure 1:**
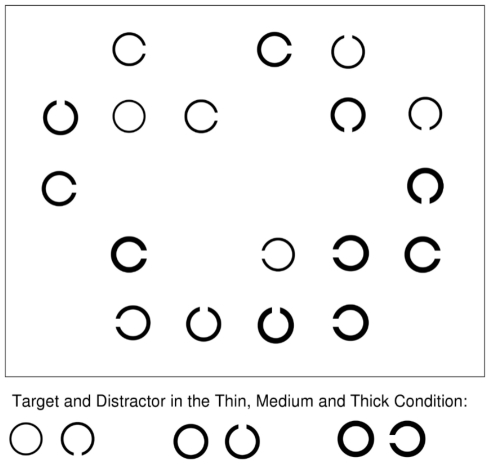
Example of the search display and the conditions. An example of a search display, depicting a target present trial in search for a thin target ring, among thin, medium and thick Landolt Cs. The Figure is not drawn to scale; the stimuli were much smaller and had a larger distance than depicted. The bottom row depicts examples of the possible target-distractor combinations used in each block.

The search display consisted of 18 black Landolt rings with a diameter of 1° that either had no gap, or a gap of 0.3° that could be oriented upwards, downwards, or to the left or right. The stimuli were presented against a white background on a regular 6×6 matrix, so that the minimum distance between stimuli was 5.7° horizontally, and 4.4° vertically (centre to centre). The 18 search items consisted of equal numbers of stimuli with thin line-width (0.05°), medium line-width (0.15°) and thick line-width (0.3°). Figure 1 depicts an example of the stimulus display.

### Design

The experiment consisted of the 3×2 within-subjects conditions “target type” (thin, medium, or thick) and “target presence” (present vs. absent). The target type was blocked, and the order of blocks was counterbalanced between participants. Target presence, position and gap orientation were varied randomly. Participants completed 180 trials per block.

### Procedure

Each trial started with a fixation control: The search display was only presented if the tracking was stable (no blinks) and the gaze was within 50 pixels (1.2°) of the centre of the fixation cross, for at least 500 ms (within a time-window of 3,000 ms). Otherwise, participants were calibrated anew (9-point calibration) and the next trial started again with the fixation control.

Upon presentation of the stimulus display, participants were required to search the display for the target, and to press the right versus left mouse button when the target was present versus absent, respectively. The stimulus display remained on screen until response, and was immediately succeeded by a feedback display (for 500 ms) consisting in the black printed words “Correct !” or “Wrong !” (Arial Black, 13 pt.), presented centrally. After an intertrial interval of 250 ms, in which a blank white screen was presented, the next trial started with the presentation of the fixation control.

Before each block, participants were given written instructions about the task and the target in the next block, but no specific instructions concerning their eye movements. The first 30 trials in each block were discounted from all analyses as practice trials.

## Results

### Data

In the RT and eye movement analyses, only correct trials with RTs below 4,000 ms were included (<0.01% of data loss). Data were analysed with repeated-measures ANOVAs and two-tailed t-tests, whereby the significance level of *p*<.05 was based on the Greenhouse-Geisser corrected *p*-values, which will be reported together with the uncorrected degrees of freedom.

Eye-movement data were parsed into saccades and fixations using Eyelink’s standard parser configuration, which classifies an eye movement as a saccade when it exceeds 30°/sec velocity or 8,000°/sec^2^ acceleration. Successive fixations on the same region (that were separated by small, corrective saccades) were counted as a single fixation, and the dwell time was summed across the two fixations. Fixations were attributed to a distractor or target when they were within 50 pixels (1.3°) of the centre of a stimulus. This restrictive fixation criterion was used in order to exclude fixations falling between stimuli from analyses (centre-of-gravity fixations), whose dwell times might be influenced by other factors than the stimulus characteristics of the most adjacent stimulus [12]. – The data were also analysed with a more liberal fixation criterion where fixations were excluded when they were more than 100 pixels away from the centre of stimulus. This however did not change the result pattern.

### RTs and Errors

The mean RTs and error scores are depicted in Table 1. A 3×2 ANOVA comprising the variables “target type” (thin vs. medium vs. thick) and “target presence” (present vs. absent) computed over the mean RTs showed that RTs were fastest with a thick target, intermediate with a thin target, and slowest when the target was medium (*F*(2,22) = 21.8; MSE = 69,743; *p*<.001). Moreover, RTs were faster on target present trials than on target absent trials (*F*(1,11) = 176.0; MSE = 60,380; *p*<.001), and these differences were significantly larger when the target was medium than when it was thin or thick (*F*(2,22) = 8.7, MSE = 15,398; *p* = .002).

**Table 1:** Mean RTs and Percentage of Errors on Target Present and Absent Trials in Each of the Three Search Conditions (Thin, Medium, and Thick, respectively).

		*Target Type*		
		Thin	Medium	Thick
RTs	*present*	1,518	1,577	1,218
	*absent*	2,220	2,517	1,882
				
Errors	*present (misses)*	9.46%	17.69%	10.71%
	*absent (false alarms)*	9.98%	4.03%	2.72%

The same analysis computed over the mean error scores showed significant main effects of the target type (*F*(2,22) = 5.1; MSE = 31.3; *p* = .03), of target presence (*F*(1,11) = 79.4; MSE = 11.2; *p*<.001) and a significant interaction between the two variables (*F*(2,22) = 11.4; MSE = 44.0; *p* = .003). The interaction was due the fact that, in the medium and thick target conditions, misses occurred significantly more frequently than false alarms (all *p*s<.001), whereas these did not differ in the thin target condition (*p* = .86). This result pattern may reflect that observers believed that they were more likely to miss the thin target than the medium or thick target and tried to compensate for the differences by adapting their response criterion (or “guesses”). More importantly, accuracy mirrored the trends found in the RTs (see Table 1), indicating that interpretation of the data is not complicated by a speed-accuracy trade-off.

### Number of Fixations

**Figure 2:**
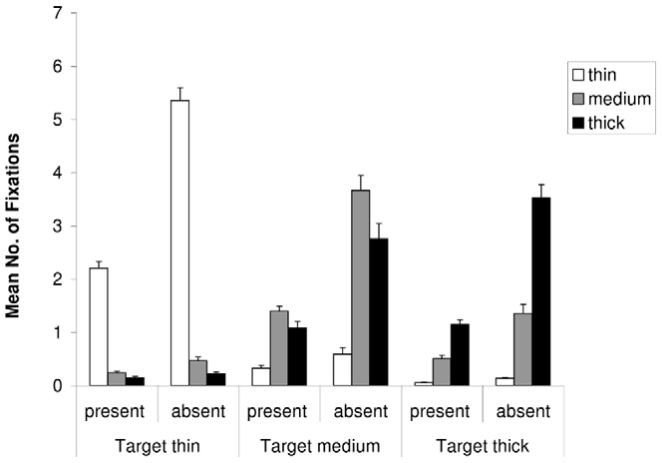
Results: Mean Number of Distractor Fixations. The mean number of fixations on each distractor type during visual search for a thin, medium or thick target, respectively. Error bars depict +1 SEM.

A 3×2×3 ANOVA comprising the variables “target type” (thin vs. medium vs. thick), “target presence” (present vs. absent), and “distractor type” (fixation on thin vs. medium vs. thick distractor) computed over the mean number of fixations per trial showed that all main effects and interactions reached significance (all *F*s>7.0; all *p*s<.005). As shown in Figure 2, fixations were clearly modulated by target-distractor similarity, with most fixations being made on distractors that were most similar to the target. Separate 2×2 ANOVAs comparing the number of fixations on two distractor types (thick vs. medium, medium vs. thin, thick vs. thin) on present versus absent trials confirmed that, in search for a thin target, thin distractors were significantly more frequently selected than medium distractors (*F*(1,11) = 435.5; *p*<.001) and thick distractors (*F*(1,11) = 429.5; *p*<.001). Moreover, the more similar, medium distractors were also more frequently selected than thick distractors (*F*(1,11) = 60.0; *p*<.001). These differences were reliable on both target absent and target present trials, but were all significantly stronger on target absent trials (all *F*s>10.1; all *p*s<.009).

Similarly, when the target was medium, medium distractors were more frequently selected than thin distractors (*F*(1,11) = 51.6; *p*<.001) and thick distractors (*F*(1,11) = 8.4; *p*<.015). Thick distractors were also more frequently selected than thin distractors (*F*(1,11) = 55.2; *p*<.001), and all these differences were again more pronounced on target absent trials than on target present trials (all *F*s>7.2; all *p*s<.021).

Finally, in search for a thick target, thick distractors were selected most frequently; significantly more often than medium distractors (*F*(1,11) = 104.2; *p*<.001) and thin distractors (*F*(1,11) = 187.0; *p*<.001). Moreover, more similar, medium distractors were also fixated more often than thin distractors (*F*(1,11) = 62.1; *p*<.001), and all of these differences were again stronger on target absent than on target absent trials (all *F*s>39.7; all *p*s<.001).

### Dwell Times

**Figure 3:**
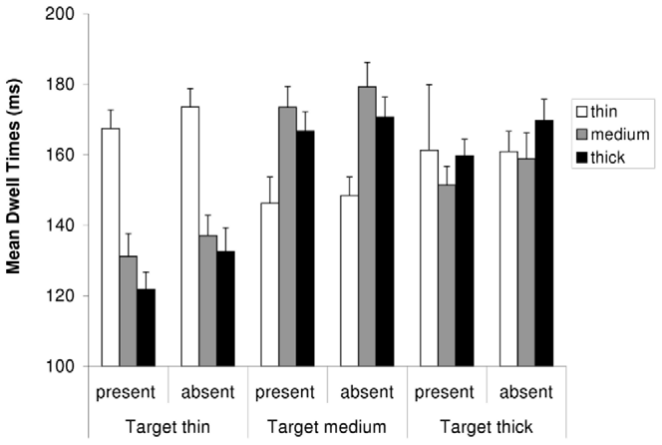
Results: Mean Dwell Times of Fixations on Each Distractor. The mean dwell times on each distractor type, depicted separately for the thin, medium and thick search target. Error bars depict +1 SEM.

The omnibus ANOVA computed over the mean dwell times (see Figure 3) showed a significant main effect of the target type (*F*(2,22) = 19.6; *p*<.001), and a significant two-way interaction between target type and distractor type (*F*(2,22) = 20.7; *p*<.001). Separate analyses revealed that, in search for a thin target, dwell times on thin distractors were longest, significantly longer than dwell times on medium distractors (*F*(1,11) = 63.7; *p*<.001) and thick distractors (*F*(1,11) = 85.3; *p*<.001), whereas dwell times on medium and thick distractors did not differ (*F*(1,11) = 2.7; *p*<.13).

In search for a medium target, dwell times on medium distractors were longest, significantly longer than on thin distractors (*F*(1,11) = 61.1; *p*<.001) and on thick distractors (*F*(1,11) = 7.4; *p* = .020). In addition, dwell times on thick distractors were also significantly longer than on thin distractors (*F*(1,11) = 38.9; *p*<.001).

When the target was thick, dwell times were only significantly longer on thick distractors than on medium distractors (*F*(1,11) = 6.2; *p* = .030), whereas dwell times on thin distractors did not differ significantly from dwell times on thick or medium distractors. The failure to find significant differences between dwell times on thin distractors and dwell times on the remaining distractor types could be due to the fact that fixations on thin distractors were very rare in search for a thick target. This is also reflected in the rather large variance in dwell times on thin distractors (see Figure 3), which presumably rendered it difficult to detect significant differences between the conditions.

This result pattern is inconsistent with the hypothesis that dwell times are predominantly determined by the perceptual difficulty of detecting the gap. On this view, dwell times on the thin distractors should have been longest across all conditions, whereas dwell times on the thick distractors should have been consistently short. Contrary to this, the results showed that dwell times on thin, medium and thick distractors strongly depended on the line-width of the target, with dwell times being longest on distractor items that were most similar to the target. This is in line with the view that dwell times on distractors vary according to their similarity to the pre-defined target [4].

To examine whether perceptual difficulty modulates dwell times when similarity is held constant, dwell times were also compared between only the similar distractors. If detection difficulty modulates dwell times on top of similarity, then we would expect dwell times to be longest on thin distractors in the thin target condition, and shortest on thick distractors in the thick target condition, with intermediate dwell times for medium distractors in the medium target condition. A 2×3 ANOVA computed over the mean dwell times on target present and absent trials of the different types of similar distractors (thin, medium, thick) yielded a significant main effect of target presence (*F*(1,11) = 19.4; MSE = 49.3; *p* = .001), with shorter dwell times on target present trials (*M* = 167 ms) than on target absent trials (*M* = 174 ms), and a significant main effect of the distractor type (*F*(2,22) = 5.7; MSE = 142.5; *p* = .010). However, contrary to the prediction above, dwell times on the medium distractor were longer (*M* = 176 ms) than dwell times on both thin and thick distractors (*M* = 170 ms and 165 ms, respectively). Separate 2×2 ANOVAs showed that only the dwell time differences between the medium and thick distractors reached significance (*F*(1,11) = 12.5; MSE = 131.0; *p* = .005; all other *p*s>.089). With this, the results mimic the differences in search efficiency, or in the number of fixations: When only dwell times on the most similar distractors are considered, dwell times apparently reflect the difficulty of discriminating the target from the distractors, and not the difficulty of identifying distractors.

## Discussion

### Summary

Previous conclusions that dwell times in visual search are determined by similarity failed to rule out perceptual difficulty [4]. In the present study, perceptual difficulty was varied independently from target similarity, by testing 3 target line-widths (thin, medium, and thick). The results supported the earlier conclusion that dwell times are primarily determined by target similarity: Dwell times were longer for distractors with the same line-width. By contrast, dwell times did not appear to be affected by line thickness per se, contrary to the view that dwell times are primarily determined by the perceptual difficulty of detecting the gap. This indicates that, in prior visual search experiments, dwell times were probably also mainly determined by the similarity of an item to the target, and not by the perceptual difficulty of the target present/absent decision.

However, this should not be taken to mean that perceptual difficulty will never modulate dwell times. Previous studies found that presenting all stimuli at low luminance contrasts reliably elongated dwell times [8]. One possible explanation for the discrepant results is that stimulus contrast may only affect dwell times in a certain range of stimulus contrasts, near the detection threshold: Research on reading has shown that stimulus contrast only modulates dwell times when the stimulus contrast is very low, and the detectability approaches the detection threshold [9]–[10] [13]–[15]. Similarly, studies which varied the gap size of Landolt C’s, or the luminance contrast of the stimuli, used very low contrasts and very small gap sizes [8] [6]–[7]. By contrast, in the present study, the stimuli were all clearly supra-threshold and could be identified quite effortlessly, which might have prevented detecting an effect of perceptual difficulty on dwell times.

### Implications for Current Models Explaining Dwell Times

It is interesting to note that, in the present study, dwell times closely followed the result pattern found in the mean number of fixations: When the target was thin, thin distractors were fixated more frequently and for longer than thick or medium distractors, and when the target was medium, medium distractors were fixated more frequently and significantly longer than the other distractors (compare Figures 2 and 3). The only exception is the thick target condition, where the differences between dwell times on thick and thin distractors were greatly diminished. However, this is probably due to the fact that there were only very few fixations on the thin distractor, resulting in an inaccurate estimate of dwell times on the thin distractors (see Fig. 2).

The number of fixations, dwell times and RTs were all highest when the target was medium. Previous studies already showed that search is less efficient for medium targets than for small or large targets [16]–[18]. The medium target was probably more difficult to find because it was somewhat similar to both thin and thick distractors, rendering target discrimination more difficult. By contrast, the thin and thick targets were similar to only one type of distractor (i.e., the medium one), so that they were better discriminable from the distractors [11] [19]. The finding that dwell times were overall longest with the medium target, and were moreover strongly affected by target similarity suggest that dwell times are determined by target discriminability, and thus by the same factors that determine search efficiency.

An important consequence of this finding is that dwell times appear to be linked to the goals of the observers (e.g., their mental representation of the target), and the time needed to actively process stimuli. Foveated distractors are probably not all processed to the same depth (e.g., until they are fully identified), but only to a degree that observers can be sufficiently sure that the foveated stimulus is not the target. Presumably, similar stimuli produce longer dwell times because they need more in-depth processing to distinguish them safely from the target. This indicates that in visual search, dwell times are linked to active processing of features which in turn proceeds on a need-to-know basis [20]. – To note, it is still possible that the stimulus is processed further after the eyes have moved over to the next stimulus so that it is fully identified in the end. However, the finding that dwell times vary with target similarity indicates that *the mechanism triggering the next eye movement* does not depend on difficulties associated with full identification of the stimulus, but depends only on the time needed to determine whether the foveated stimulus is a distractor or the target.

With this, the present results are more in line with *process montitoring models*, which propose that dwell times are mostly determined by the time needed to process the fixated stimulus to a certain stage [14] [21], than with *global estimation models* that assume that dwell times are based on estimates of the required processing, derived from the prior fixation(s) or the previous trial(s) [6] [9]. The demonstration that dwell is dependent on target-distractor similarity means that global estimates about the required dwell time do not completely determine dwell times. Previous failures to find a more direct connection between the properties of the foveated stimulus and dwell times may be rooted in the failure to systematically vary the target similarity of the distractors in mixed displays that do not allow predicting the required dwell time from the previous fixation [22]. This possibility has to be investigated in further studies.
